# The Effect of Exposure to Mobile Phones on Electrical Cardiac Measurements: A Multivariate Analysis and a Variable Selection Algorithm to Detect the Relationship With Mean Changes

**DOI:** 10.1155/2024/7093771

**Published:** 2024-10-03

**Authors:** Nader Alharbi, Mohammed Alassiri

**Affiliations:** ^1^ Department of Basic Sciences College of Science and Health Professions King Saud bin Abdulaziz University for Health Sciences (KSAU-HS), Riyadh, Saudi Arabia; ^2^ King Abdullah International Medical Research Centre (KAIMRC), Riyadh, Saudi Arabia; ^3^ Pathology and Laboratory Medicine King Abdulaziz Medical City (KAMC), Riyadh, Saudi Arabia

**Keywords:** ECG, electromagnetic field, mobile phone, multivariate analysis

## Abstract

**Background:** The exponential growth in mobile phone usage has raised concerns about electromagnetic field (EMF) exposure and its health risks. Blood pressure and BMI, which impair heart function due to decreased adrenoreceptor responsiveness, parasympathetic tone withdrawal, and increased sympathetic activity, may further exacerbate these risks. However, the effects of radiofrequency electromagnetic (RF-EM) exposure from mobile phones on electrocardiograms (ECGs) and heart rate variability (HRV) in individuals remain unclear.

**Purpose:** Building upon our previous findings on HRV changes due to mobile phone proximity, this study is aimed at significantly enhancing the analytical approach used to assess the effects of mobile phones on cardiac parameters. This study exploits data from a previous study but with a different purpose. The aim of this study is twofold: (a) to examine whether exposure to mobile phones changes the five variables (P-R, QRS, QT, ST, and HR) in a multivariate manner and (b) to examine whether the blood pressure and/or the body mass index (BMI), which acts as a proxy for obesity, have an effect on the change of these five variables. For both aspects of the study, four cycles are performed.

**Method:** We conducted multivariate analysis on previously collected electrical cardiac measurement data from 20 healthy male subjects exposed to mobile phone EMF, with the mobile phones placed at four different body locations. The one-sample Hotelling *T*^2^ test on the mean vector of differences was utilised instead of multiple paired *t*-tests. This multivariate method comprehensively analyzes data features and accounts for variable correlations, unlike multiple univariate analyses. Given our small sample size, we employed the MMPC variable selection algorithm to identify predictor variables significantly related to mean changes.

**Results:** Significant alterations in ECG intervals and heart rate were noted in the subjects before and after the first EMF exposure cycle, independent of their BMI. Notably, heart rate, P-R, and QRS intervals fell postexposure while QT and ST intervals increased. These changes were influenced by variations in systolic blood pressure, with BMI showing no significant effect.

**Conclusion:** The observed modifications in cardiac electrical measurements due to mobile phone EMF exposure are attributed to the effects of EMF itself, with no impact from BMI on the extent of these changes.

## 1. Introduction

Addictive use of mobile phones can lead to a variety of physical and mental health issues, such as anxiety, depression, sleep disturbances, and even impairments to cognitive functions. Research has also linked mobile phone addiction to problematic behaviors, such as compulsive gambling and internet overuse [1]. To help prevent such addiction, daily usage limits should be monitored. The World Health Organization (WHO) recommends that people limit their exposure to electromagnetic field (EMF) radiation, such as that emitted from mobile phones, to levels that are as low as reasonably achievable [2].

While mobile phones offer countless benefits, they also come with a range of potential risks that are often overlooked. One of the main risks associated with them is the potential for exposure to electromagnetic radiation, including radiofrequency (RF) and EMF [3]. Studies have shown that prolonged exposure to RF-EMF can cause a range of adverse health effects, including increased risk of certain types of cancer, decreased fertility, and neurological problems [3, 4]. Furthermore, research has also suggested that children and adolescents may be particularly susceptible to the effects of RF-EMF, as their bodies are still developing and their skulls are thinner than those of adults [2, 5]. Additionally, the literature shows that there is an association between the magnitude of damage caused by EMF and the duration of exposure; evidence for this is that long-term exposure may cause changes at a cellular level [6, 7]. For example, it can disrupt normal functioning within the body's organs, particularly those related to cardiovascular health such as the heart muscle itself or its electrical system responsible for controlling heartbeat rate and rhythmicity [6–8].

Research also suggests that individuals with prolonged exposure to mobile phone radiation may be more likely than those without such exposure to palpitations and chest pain symptoms related to cardiac arrhythmia [6, 7]. Other studies have suggested similar links between long-term mobile phone use and increased risk of cardiovascular disease (CVD) due primarily to oxidative stress caused by excessive free radical production, as well as changes in blood pressure (BP) regulation [9].

There is a growing body of evidence linking EMF and autonomic function disturbances in the heart [8, 10, 11]. This could potentially lead to serious health complications such as arrhythmias or even sudden cardiac death for those exposed to high levels of radiation from their mobile phones for extended periods of time. Furthermore, research suggests that EMFs may be responsible for an increased risk of coronary artery disease and stroke due to their effects on arterial stiffness [10, 12, 13].

The impact of mobile phone radiation on cardiac health and function remains a concern. The beat-to-beat variation in heart rate (HR) is referred to as heart rate variability (HRV) and is a reliable indicator of the heart's autonomic control. In long-term research, reduced HRV has been linked to an increased risk of CVD and mortality [14]. Exposure to an EMF considerably reduces HRV while increasing the HR and sympathetic activity [15]. It has also been found that healthy, age-matched people who had used their mobile phones for more than 10 years had a drop in HRV, which was associated with an increase in sympathetic activity [16]. In contrast, adolescent students exposed to RF-EM for a brief period had a lower HR and enhanced parasympathetic activity [17]. Numerous investigations, however, have found no correlation between RF-EM exposure and BP, HR, or HRV [18–22]. Another important indicator of heart function is the ECG, which indicates the cardiac signal; it has been shown that EMF influences the ECG [23]. Moreover, long-term exposure to EMF can lead to heart problems by changing the duration of ECG intervals [24].

By examining the ECG data of individuals with a significant history of mobile phone use, this research is aimed at shedding light on how EMF exposure may affect cardiac health. This inquiry is critical for informing public health strategies and enhancing understanding of the interactions between technology use and cardiovascular well-being.

In addition to the research questions, this work employs a more sophisticated statistical analysis method compared to [15]. While [15] relied on univariate analysis, specifically *t*-tests between groups for each variable, this study advances the methodology by using multivariate tests. This approach analyzes all variables simultaneously when comparing the two groups (before and after exposure). The benefit of multivariate testing is twofold. First, it allows for a single, comprehensive test instead of multiple individual tests. Second, it strengthens the statistical analysis by considering potential correlations between the variables. Furthermore, variable selection was conducted to identify whether BP (systolic and diastolic) and BMI influence the five outcome variables. This step helps to gain a deeper understanding of how these factors affect the five variables.

The use of multivariate tests in this study is pivotal in identifying and understanding the complex interdependencies and potential interactive effects among the cardiac parameters. This methodological enhancement is critical for overcoming the limitations of univariate analyses, which may overlook subtle yet significant patterns of change in cardiac function due to mobile phone exposure. By leveraging these advanced statistical tools, the study is aimed at providing a more comprehensive and accurate understanding of the physiological impacts of mobile phone use, facilitating the development of more informed health guidelines and preventative measures against potential adverse effects.

In the following sections, we detail our methodology, including subject selection and experimental procedures, followed by a statistical analysis to assess the effect of EMF exposure on heart function. We conclude with a discussion of the implications of the findings for health strategies and of our understanding of EMF on the cardiovascular system.

## 2. Materials and Method

### 2.1. Ethical Statements

The study has been reviewed and approved by the Institutional Review Board (number SP17/064/R) at King Saud bin Abdulaziz University for Health Sciences (KSAU-HS). All participants agreed voluntarily and signed a consent form.

### 2.2. Study Subjects

**Figure 1:**
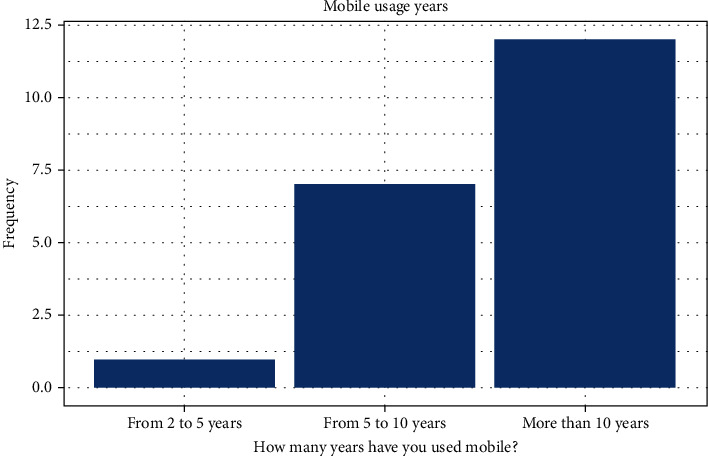
Mobile phone usage time.

Twenty healthy male subjects were recruited from the College of Medicine at KSAU-HS and divided based on their weight. The students had an average age of 23 ± 2 years and a BMI of 28.20 ± 5.99. All the participants were briefed about the procedure, and consent forms were distributed. To facilitate better data analysis, mobile phone usage time was recorded for all the participants (see Figure 1).

To establish accurate baseline readings, all the participants were asked to refrain from consuming caffeinated drinks or performing physical activities. Additionally, they were required to abstain from using their mobile phones for 12 h before the study. On the day of the experiment, they were familiarized with the laboratory examination room, experimenters, equipment, and protocols. They also underwent a brief physical examination by a health professional from the college to ensure their physical well-being. All the subject characteristics were recorded, as shown in Table 1. Any subjects using medication or suffering from chronic debilitating diseases were excluded from the study. Furthermore, any participant displaying signs of academic or social stress during the briefing was also excluded.

### 2.3. Experimental Procedure

The study design was adapted from existing literature; however, we extended the recording time and incorporated a 15-min recovery period between recordings. During this time, participants were escorted outside the lab, and their mobile phones were switched off. The study took place in the physiology lab at COM. All personal mobile phones were left outside the lab, except for the one used in the study (an iPhone 5 Plus). Moreover, the participants were asked to remove any metal objects, such as rings or belts, to prevent signal interference. Before attaching the electrodes, we recorded and evaluated physical signs and BP. We then commenced ECG recordings over a 2-h period using a three-electrode recording device (Lead II) on a PowerLab 8/30 ML870 high-performance data acquisition system (ADInstruments, Dunedin, New Zealand). ECG readings were captured across four cycles, varying by the mobile phone location on the participant's body.

**Figure 2:**
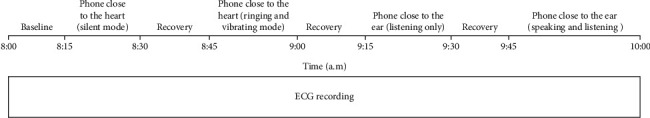
Depiction of the experimental procedure [15].

The first cycle involved placing the mobile phone in silent mode in a pocket near the heart of the participant. For the second cycle, the ringing and vibrating pocket point, the mobile phone was placed in the same location but was set to active (ringing and vibrating) mode. In the third cycle, the ear point, the mobile phone was positioned near the participant's ear, who was then asked to listen to a mobile phone conversation. The fourth cycle was similar to the third, except the participant also spoke using the mobile phone. All ECG recordings were conducted during working hours from 08:00 am to 16:00 pm, with each session lasting 15 min (see Figure 2).

### 2.4. Data

The ECG data were analysed using LabChart 7.1 software. They were examined over a 5-min period, which is considered to be the optimal duration for extracting HRV spectrum studies, as recommended by the European Society of Cardiology Task Force and the North American Society of Pacing and Electrophysiology. To minimise false ECG readings attributable to participants' stress, the first 5 min of all the ECG recordings were excluded from the analysis. They were analysed beat-by-beat for ECG intervals and in milliseconds for P-R, QRS, ST, and QT intervals. In addition, HR was calculated from the signals using the software's ECG module.

## 3. Statistical Analysis

The repeated measurement approach facilitates hypothesis testing concerning the state of exposure. The initial hypothesis aimed to assess the equality of the mean vectors of the five variables across each cycle, determining whether there were any differences in these before and after exposure. Rather than conducting multiple paired *t*-tests, we utilised the one-sample Hotelling *T*^2^ test on the mean vector of differences. This multivariate approach provides a comprehensive analysis by considering the features of the data in their entirety, unlike the univariate approach. Additionally, it accounts for correlations among variables, an aspect overlooked by multiple univariate analyses.

The MMPC variable selection algorithm [25] was employed to identify which of the three predictor variables were statistically significant in relation to the mean changes. Given the small sample size, this algorithm, designed specifically for such scenarios, was particularly suited to our analysis.

The statistical analysis was performed in the statistical software *R* [26], and the packages *Rfast* [27], *Compositional* [28], and *MXM* [29] were utilised.

### 3.1. Results

We conducted an analysis of repeated measurements from the 20 subjects, divided into four cycles, focusing on five essential variables per subject: P-R, QRS, QT, ST, and HR. These variables were measured both before and after exposure to examine their potential variations.

**Figure 3:**
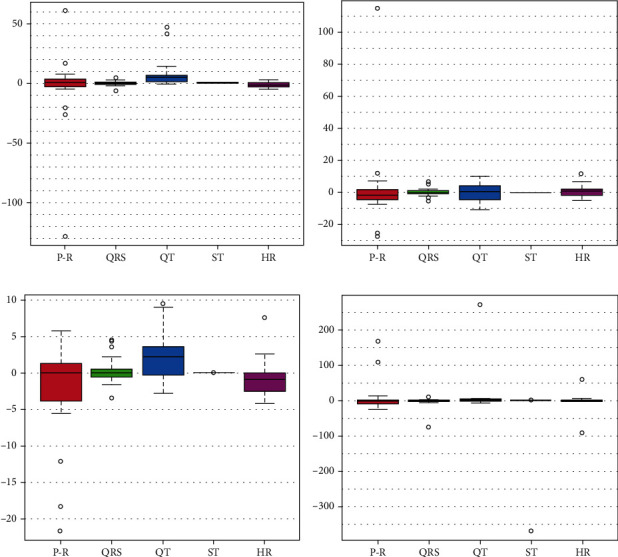
Box plots of the differences in the variables between before and after exposure: (a) first cycle, (b) second cycle, (c) third cycle, and (d) fourth cycle.

Figure 3 shows the differences in measurements before and after exposure across all four cycles. Despite the overall differences being minimal, indicating stable measurements, outliers were noted in all cycles, indicating both positive and negative deviations. Notably, the third cycle primarily exhibited negative outliers.

Our statistical evaluation, detailed in Table 2, applied the Hotelling *T*^2^ test to compare means before and after exposure. The findings reveal no statistically significant differences in three of the four cycles, with the first cycle showing a notable mean difference (*p* value = 0.01). Thus, it can be said that the exposure does not change, statistically significantly, between before and after three cycles.

Further examination sought to explore the relationship between changes in mean vectors postexposure and subjects' BP (both systolic and diastolic) and BMI. The MMPC variable selection algorithm facilitated the identification of statistically significant predictors among the small sample size, as reported in Table 3. This methodology diverges from the previous study [15] by employing a multivariate test for mean vector analysis, enhancing the robustness of the findings beyond the scope of multiple univariate *t*-tests. Furthermore, the precise identification of significant predictors using the MMPC algorithm marks a substantial methodological advancement in our research.

The variable selection algorithm showed that there is a link between the mean vector change in the first cycle and systolic BP. This indicates that the systolic BP affects the exposure, but only for the first cycle. The diastolic pressure is associated with the mean changes of the exposure, but only for the third cycle. Finally, none of these two aforementioned factors, not the BMI, are associated with the mean change in the variables during the exposure for the second and fourth cycles.

## 4. Discussion

The primary aim of the study was to employ hypothesis testing to examine the equality of mean vectors across five variables in each cycle, thereby assessing any differences in measurements before and after exposure to EMF. A multivariate analysis was performed on ECG and HRV parameter data before and after exposure to EMF, uncovering a statistical difference in the ECG data during the first cycle (first exposure to EMF) when the phone was positioned near the heart (Table 1).

These findings are intriguing and consistent with a substantial body of evidence indicating a significant impact of EMF on ECG [22]. The multivariate analysis suggests that the observed alterations were attributable to the EMF emitted by mobile phones. Furthermore, Table 3 reveals that the variations in mean vectors before and after exposure are associated with the subjects' BP (both systolic and diastolic). The table indicates a probable connection between the changes in mean vectors and systolic BP during the initial cycle. Additionally, the *p* value for assessing the impact of BMI on the mean changes before and after ECG exposure proved to be insignificant, reinforcing the study's objective. Hence, the effects of mobile phone EMFs on the body are consistent, irrespective of an individual's BMI, highlighting the necessity for monitoring EMFs.

Regarding the clinical implications of the changes in ECG intervals, numerous experimental studies have determined that the impact of EMF on ECG data will be in the form of changing the duration of the different ECG intervals, for example, prolonging some intervals [30]. Our research observed a notable reduction in the P-R, QRS, QT, and ST intervals, as well as in HR. This finding aligns with observations reported by other studies [15, 30, 31]. Furthermore, there is an association between variations in ECG intervals and CVD in humans [32]. More specifically, they investigated the impact of mobile phone EMF on the ECG of ischemic heart disease patients, and they showed that EMF indeed influences the ECG intervals by prolonging QT intervals [31]. As a result, exposure to mobile phone EMFs should be examined carefully, especially among people with heart conditions.

## 5. Conclusion

Given the evidence linking EMFs from mobile phones to adverse effects on heart health, it is crucial for regular users of such devices, especially those at risk of CVDs, to take steps towards reducing their overall exposure. This can be achieved through measures such as limiting talk time or using hands-free headsets during calls. Additionally, ongoing research in this area is essential to deepen our understanding of how these potentially harmful frequencies impact our bodies over both short- and long-term periods.

## Figures and Tables

**Table 1:** Characteristics of the subjects. Values are presented as mean ± SD.

*N*	20
Age	23 ± 2
Systolic (mmHg)	115.06 ± 11.45
Diastolic (mmHg)	71.06 ± 10.59
Weight (kg)	85.43 ± 20.42
Height (m)	1.74 ± 0.06
BMI (kg/m^2^)	28.20 ± 5.99

**Table 2:** Mean vectors of the five variables for each cycle and exposure. The last column reports the Hotelling *T*^2^ test *p* values for the equality of means before and after exposure.

**Cycle**	**Exposure**	**Variables**					**p** ** value**
		**P-R**	**QRS**	**QT**	**ST**	**HR**	
1st	Before	176.855	85.301	325.360	0.049	74.038	**0.01**
	After	172.405	85.243	333.535	0.052	72.412	
2nd	Before	170.895	84.724	328.553	0.053	71.853	0.08
	After	168.395	85.164	330.847	0.053	71.143	
3rd	Before	171.830	86.085	330.830	0.052	71.372	0.165
	After	174.315	86.024	330.830	0.055	72.091	
4th	Before	167.697	89.690	317.884	18.411	72.841	0.789
	After	178.300	85.880	331.090	0.055	71.869	

*Note*: *p* value with bold emphasis is significant.

**Table 3:** *p* values for testing the effect of blood pressure and BMI on the mean changes before and after exposure.

**Cycle**	**Predictor variables**		
	**Systolic blood pressure**	**Diastolic blood pressure**	**BMI**
1st	**0.005**	0.106	0.32
2nd	0.473	0.656	0.150
3rd	0.540	**0.008**	0.643
4th	0.860	0.929	0.792

*Note*: Values with bold emphasis are significant.

## Data Availability

All data used, generated, or analysed during the study are available upon request.
